# Rapid Development of New Protein Biosensors Utilizing Peptides Obtained via Phage Display

**DOI:** 10.1371/journal.pone.0024948

**Published:** 2011-10-07

**Authors:** Jun Wu, Jong Pil Park, Kevin Dooley, Donald M. Cropek, Alan C. West, Scott Banta

**Affiliations:** 1 Department of Chemical Engineering, Columbia University, New York, New York, United States of America; 2 United States Army Engineer Research and Development Center, Construction Engineering Research Laboratory (CERL), Champaign, Illinois, United States of America; University of Houston, United States of America

## Abstract

There is a consistent demand for new biosensors for the detection of protein targets, and a systematic method for the rapid development of new sensors is needed. Here we present a platform where short unstructured peptides that bind to a desired target are selected using M13 phage display. The selected peptides are then chemically synthesized and immobilized on gold, allowing for detection of the target using electrochemical techniques such as electrochemical impedance spectroscopy (EIS). A quartz crystal microbalance (QCM) is also used as a diagnostic tool during biosensor development. We demonstrate the utility of this approach by creating a novel peptide-based electrochemical biosensor for the enzyme alanine aminotransferase (ALT), a well-known biomarker of hepatotoxicity. Biopanning of the M13 phage display library over immobilized ALT, led to the rapid identification of a new peptide (ALT5-8) with an amino acid sequence of WHWRNPDFWYLK. Phage particles expressing this peptide exhibited nanomolar affinity for immobilized ALT (K_d,app_ = 85±20 nM). The newly identified ALT5-8 peptide was then chemically synthesized with a C-terminal cysteine for gold immobilization. The performance of the gold-immobilized peptides was studied with cyclic voltammetry (CV), QCM, and EIS. Using QCM, the sensitivity for ALT detection was 8.9±0.9 Hz/(µg/mL) and the limit of detection (LOD) was 60 ng/mL. Using EIS measurements, the sensitivity was 142±12 impedance percentage change %/(µg/mL) and the LOD was 92 ng/mL. In both cases, the LOD was below the typical concentration of ALT in human blood. Although both QCM and EIS produced similar LODs, EIS is preferable due to a larger linear dynamic range. Using QCM, the immobilized peptide exhibited a nanomolar dissociation constant for ALT (K_d_ = 20.1±0.6 nM). These results demonstrate a simple and rapid platform for developing and assessing the performance of sensitive, peptide-based biosensors for new protein targets.

## Introduction

**Figure 1:**
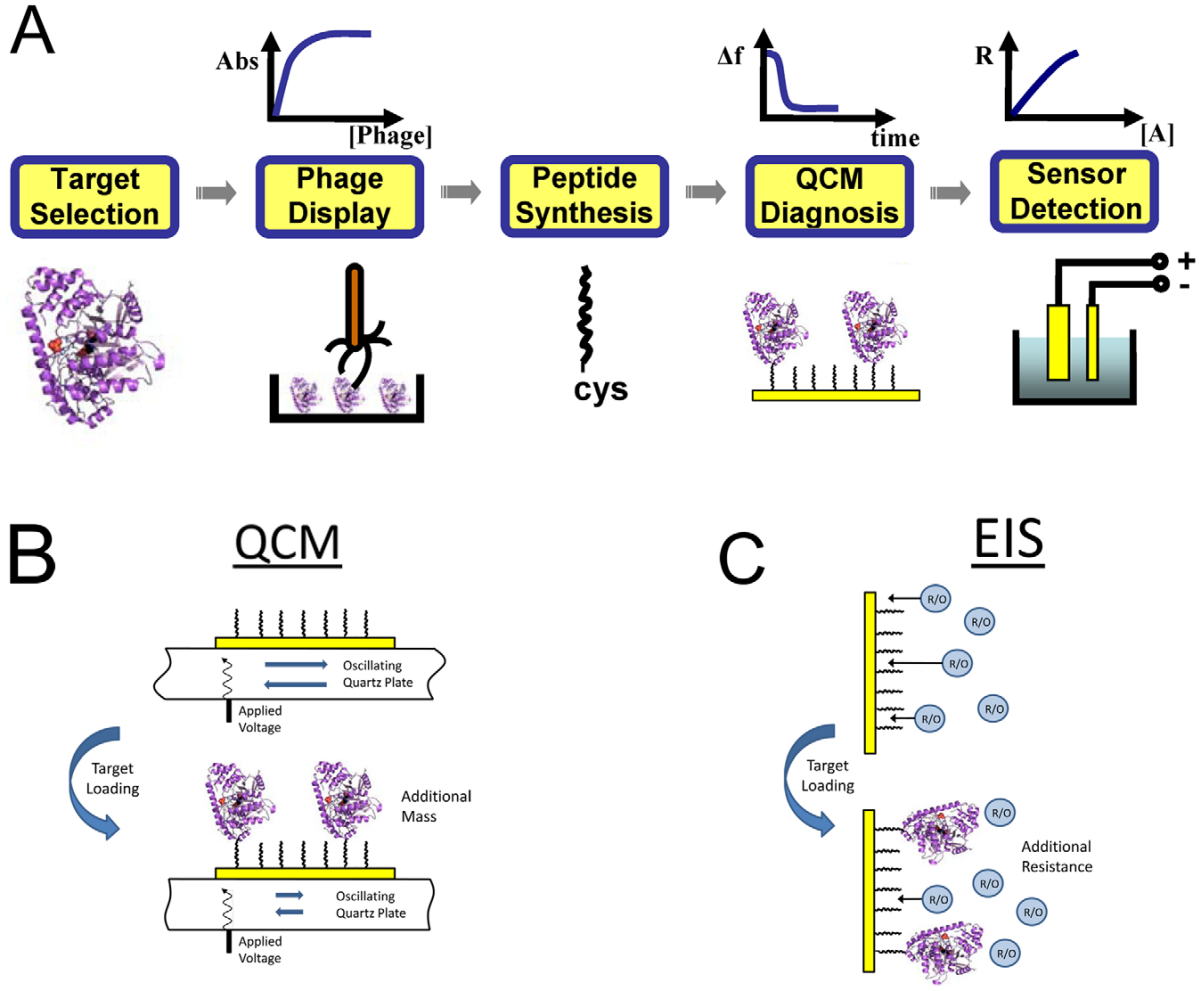
Schematic diagram illustrating the general process of biosensor development. (A) Work flow diagram for biosensor development: 1. Target protein selection, 2. Phage display selection, 3. Peptide synthesis, 4. QCM diagnosis, 5. Biosensor detection. B) The basic principle of QCM where the binding of the target protein to the immobilized peptides causes a frequency change in the oscillation of the quartz crystal. C) The basic principle of EIS where the binding of the target protein to the immobilized peptides causes increased resistance to the reaction of an added redox couple. See text for details.

A biosensor is an analytical device that combines a recognition element with a transducer (detection element) for the detection of a biological analyte (target) [1], [2]. The recognition process utilizes the affinity of the recognition element to the analyte and the interaction information is transmitted as a measurable signal (electrical, optical, etc.) by the transducer. The overall selectivity and the sensitivity of the biosensor are dependent on both the recognition element and the transducer. In this work, we demonstrate a general pathway for the development of new biosensors utilizing unstructured peptides selected using M13 phage display as the recognition element, QCM as a diagnostic tool during development, and electrochemical techniques (CV, EIS) as the detection elements. This procedure is fast and can be applied to almost any desired protein target (Figure 1A).

Immunosensors are commonly used biosensors that rely on antibodies as the biomolecular recognition element and require multi-step processing and labeling of the samples [3]. The widespread use of antibody-based immunoassays has been hindered by their high cost and the significant time necessary to develop new antibodies to emerging targets [4]. Several efforts have been made to address these limitations on the biomolecular recognition element, including the use of nucleic acid-based aptamers [5] alternative protein scaffolds [6], and short unstructured peptides [4], [7]. Compared to more complex protein-based affinity scaffolds, short unstructured peptides have several potential advantages that can be exploited for biosensor development: 1) peptides are stable and resistant to harsh environments, 2) peptides can be synthesized easily and inexpensively, and 3) peptides can be more amenable than antibodies to engineering at the molecular level [8], [9], [10]. In addition, the immobilization of short peptides on gold electrodes for use as the recognition element has been well characterized since the early 80's [11], [12].

Biopanning of phage displayed peptide libraries is a widely utilized method that allows for the rapid selection of peptides that bind to desired protein targets. Several groups have reported biosensors where the entire phage particles from these selections (featuring multiple copies of the peptides) are employed as the sensing probes in the biosensors [13], [14], [15]. Although phage display has been widely applied to identify peptides or proteins with selective binding capabilities, the application of free (non-phage-bound) peptides in the development of biosensors has been less frequently reported. Using free peptides can be advantageous as this can simplify the electrochemical detection techniques. As an example of this approach, we have recently described a new biosensor for the detection of troponin I (a cardiac biomarker) using peptides isolated by M13 phage display [16], [17].

The QCM has been widely used in biosensor development because it is a label-free technique [18], [19], [20]. The QCM is based on a piezoelectric material (quartz), where an alternating electrical field across the quartz creates an alternating shear motion of the crystal. Through appropriate circuitry, the change in the resonance frequency, *Δf*, of the crystal is tracked in real time. At constant temperature, this resonant frequency responds primarily to the change in mass associated with the crystal surface, *Δm*, and changes in the viscoelastic properties of the fluid adjacent to the crystal (Figure 1B). The relationship between *Δf* and *Δm* is linear according to the Sauerbrey equation [16].

Electrochemical techniques can also be used for the creation of label-free biosensors [21], [22], [23], [24]. They have the potential to be inexpensive, fast responding, and low maintenance. The most well-known example of an electrochemical biosensor is the commercial glucose sensor for diabetes [25], [26], where the reaction product of glucose oxidase with glucose converts ferricyanide to the electroactive species ferrocyanide that is monitored electrochemically. Other applications also benefit from the fact that biosensing electrodes can be easily integrated into microfabricated systems and used as portable test tools for field measurements [27], [28].

Cyclic voltammetry (CV) and electrochemical impedance spectroscopy (EIS) are two frequently used techniques in these applications. Both techniques measure either the current or the impedance obtained by varying the applied potential (voltage). When applied here, the method detects binding of an analyte on the surface of an electrode by the amount it hinders the access of an electroactive redox couple (ferri/ferrocyanide, in this case) to the electrode, therefore decreasing the redox current in CV or increasing impedance in EIS. CV provides rapid evidence for binding but it is less quantitative. In EIS, the sinusoidal current obtained in response to small amplitude, sinusoidal perturbations of the potential is measured. The current response has an amplitude relative to the potential amplitude and is also out of phase from the perturbation. For this reason, results are reported as complex impedance. It is common to plot EIS data in a Nyquist plot, and fit them using an equivalent circuit model to extract the parameter of interest. Here it is the resistance of charge transfer of the redox couple, which is impacted by bound analyte (Figure 1C).

In this paper, we describe a novel biosensor for the detection of alanine aminotransferase (ALT, EC 2.6.1.2, also called glutamate pyruvate transaminase, GPT). ALT catalyzes the reversible transamination of L-alanine and α-ketoglutarate to pyruvate and L-glutamate with pyridoxal 5′-phosphate as a coenzyme. ALT is found mainly in the liver, but is also found in red blood cells, heart cells, muscle tissue and other organs, such as the pancreas and kidneys [29], [30]. Serum ALT levels are an indicator for liver damage and its detection is considered the gold standard biomarker of hepatotoxicity [30], [31], [32]. The normal concentration of ALT in blood serum ranges from 5–35 U/L (0.1–0.7 µg/mL), [29] and serum ALT levels increase up to 50-fold in connection with a variety of liver conditions, including viral infection, cirrhosis, non-alcoholic steatohepatitis, and drug toxicity [31], [33], [34]. Most ALT biosensors are based on the detection of the enzymatic activity of the ALT enzyme as opposed to the detection of the protein itself [29], [35], [36], [37].

In this article we present the selection of ALT binding peptides from an M13 phage displayed peptide library and the characterization of the binding affinities of the peptides. Once the most promising peptide was selected, a cysteine-modified free peptide was synthesized and transferred to the biosensor platform. The binding activity was monitored *in situ* by QCM, and the peptide-modified gold electrode was used to detect ALT quantitatively using EIS. The general approach (Figure 1) can be extended to develop biosensors for a wide variety of target analytes. The methodology can be easily applied in a relatively short period of time at low cost.

## Materials and Methods

### Materials

All reagents used were analytical grade or higher unless otherwise stated. *Escherichia coli* strain ER2738, a host for M13 phage, and a polyvalent M13 phage display kit (Ph.D.-12) which contained random 12-mer peptides were obtained from New England Biolabs (Ipswich, MA). ALT (human liver) and lactate dehydrogenase (LDH) were obtained from Lee Biosolutions, Inc. (St. Louis, Missouri). Horseradish peroxidase (HRP) conjugated anti-M13 monoclonal antibody was from GE Healthcare (Piscataway, NJ). Tween 20, 2,2′-Azino-bis(3-ethylbenzothiazoline-6-sulfonic acid) diammonium salt (ABTS), streptavidin (SA) from *Streptomyces avidinii*, bovine serum albumin (BSA), L-cysteine hydrochloride (98%) and all other chemicals were purchased from Sigma-Aldrich (St. Louis, MO). Polystyrene microplates were from Pierce Biotechnology (Rockford, IL). The ALT5-8 peptide modified with a C-terminal cysteine (sequence: WHWRNPDFWYLKC) was synthesized (>90% purity) by Genscript (Piscataway, NJ). Quartz crystals (AT-cut, 5 MHz) with Au deposited on both sides were obtained from Inficon (East Syracuse, New York). Buffer solutions (pH = 7.3) containing 0.01 M sodium phosphate and 0.1 M NaCl were used to make all protein (1–10 µg/mL) and peptide (0.1 mM) solutions for the QCM and EIS measurements. All solutions were prepared using Milli-Q water.

### Biopanning of Phage-Displayed Peptide Library

ALT (2.6 µg/mL) was dissolved in NaHCO_3_ buffer (100 mM, pH 8.3) and transferred to the wells of polystyrene microplates. After overnight incubation with mild agitation at 4°C, the well surfaces were coated with ALT. The well surfaces were then blocked with blocking buffer (0.1 M NaHCO_3_ containing 2% BSA and 0.02% of NaN_3_) for 1 hr at 4°C, and washed 6 times with TBST (50 mM Tris-HCl pH 7.5, 150 mM NaCl, 0.1% Tween 20) to remove weakly bound ALT. The Ph.D.-12 phage displayed random peptide library (1.5×10^11^ pfu) in 100 µL of TBS buffer (50 mM Tris-HCl pH 7.5, 150 mM NaCl) was added to the ALT coated wells, and the plate was shaken gently for 1 h at room temperature. Wells were washed 10 times with TBST to remove unbound phage. The bound phage were eluted with 100 µL of 0.2 M glycine-HCl (pH 2.2) and the elution was immediately neutralized with 15 µL Tris-HCl (1 M, pH 9.1) to prevent phage killing. The eluted phage were amplified in *E. coli* ER2738 and the phage were harvested by precipitation with NaCl/polyethylene glycol (20% (w/v) polyethylene glycol-8000 with 2.5 M NaCl). This biopanning process was repeated for five rounds. The concentration of Tween 20 in the washing step was increased from 0.1% in the first round of panning to 0.3% in the second round of panning and 0.5% in the final three rounds of panning. After each round of panning, the phage were titered using Luria-Bertani (LB) broth plates containing isopropyl β-D-thiogalactopyranoside (IPTG) and X-gal. After the fifth round of selection, twelve blue monocolonies were randomly picked and amplified for DNA sequencing.

### ELISA for Peptide Binding to ALT

To test whether or not the selected phage clones from the biopanning procedure could bind to ALT, ELISA assays were performed. Microplates were coated with ALT (2.6 µg/mL) overnight at 4°C, blocked with blocking buffer, and washed 6 times with TBST solution (containing 0.1% Tween 20). Varying amounts of amplified phage clones were added to each well and incubated for 1 hr at room temperature. After washing, HRP-conjugated anti-M13 monoclonal antibody (diluted 1∶5000 in blocking buffer) was added and incubated at room temperature for 1 hr. Following incubation and washing again with the same buffer, ABTS and H_2_O_2_ (30%, v/v) were added and the absorbance was measured after a 1 hr incubation with mild agitation at 405 nm with a microplate spectrophotometer (Molecular Devices, Sunnyvale, CA).

In order to estimate the apparent dissociation constant for the ALT5-8 clone, further ELISA experiments were performed with extended phage concentrations. The data were fit to a Langmuir isotherm using non-linear regression (SigmaPlot, San Jose, CA) and the K_d,app_ was determined.

### Kinetic Studies for Measuring Peptide Inhibition of the Enzyme

ALT enzyme kinetics were measured to determine the impact of the ALT5-8 peptide on the activity of the enzyme. Kinetic experiments were performed in a coupled assay where the pyruvate generated by ALT was reduced to lactate by LDH with the concomitant oxidation of NADH. The depletion of NADH was monitored by following its absorbance at 340 nm in UV-transparent microplates. L-alanine and α-ketoglutarate concentrations were varied from 2.5 to 10 mM and 0.5 to 10 mM respectively. Initial concentrations of pyridoxal 5′-phosphate, NADH, and LDH were 0.1 mM, 0.25 mM and 47 U/L respectively. The reactions were initiated by the addition of 330 U/L ALT and all measurements were made in triplicate. All stock solutions were prepared in 100 mM Tris-HCl buffer (pH 7.8), and all kinetic trials were conducted 300 µL at 37 °C.

The kinetic rates were fit to the bi-bi ping-pong mechanism (Eqn. 1), to obtain estimates for k_cat_ and the Michaelis constants (K_M_) for L-alanine (A) and α-ketoglutarate (B). 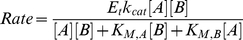
(1)


In order to determine if the peptide inhibited the enzyme as well as the nature of the inhibition, the kinetics were repeated with varying L-alanine concentrations at a constant concentration of α-ketoglutarate (1 mM). Free ALT5-8 peptide inhibitor (I) was added to the reactions at concentrations from 0–0.25 µM. The rates plotted on a Lineweaver-Burk plot to understand the mode of inhibition and the rates were also fit to the bi-bi ping-pong equation with a competitive inhibitor (Eqn. 2): 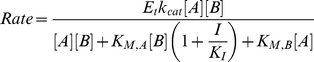
(2)


### QCM measurements

The QCM system consisted of a phase lock oscillator (PLO-10i, Maxtek, East Syracuse, New York) and a frequency counter (53131A, Agilent Technologies, Santa Clara, CA). The operation of the setup was described previously [16]. Typically, a stable baseline was achieved 1 hr after flow injection on a clean crystal at a flow rate of 50 µL/min. After a stable baseline was obtained in buffer, a peptide solution in the buffer was flowed to the cell. Usually an abrupt change in the signal occurs with the valve switching. In about 5 min, the new solution arrived at the cell and caused a frequency change due to peptide binding to the crystal surface. After the binding reached a steady state, buffer rinse was then applied and no further change in frequency was observed. Similar operations were performed for the addition of the cysteine and ALT solutions, as well as solutions of other protein competitors such as BSA and SA. All experiments were repeated three times and similar results were obtained.

### Electrochemical measurements

All electrochemical measurements were performed with a µAUTOLAB potentiostat (Type III, FRA2, Metrohm Autolab, Netherlands). A gold wire (dia. 1 mm) sealed in epoxy was used as a working electrode and a platinum mesh was used as a counter electrode. All potentials measured are reported versus a Ag/AgCl reference electrode. Both cyclic voltammetry and EIS measurements were conducted in a solution of 1 mM ferro/ferricyanide in 0.1 M sodium perchlorate.

CV and EIS were performed as previously described [16]. Briefly, the gold electrodes were prepared in 0.1 mM peptide solution for 1 hr, followed by backfilling in 1 mM cysteine solution for 2 hr to obtain a Au-Pep-Cys electrode. The Au-Pep-Cys electrodes were incubated in ALT solution for 1 hr before CV and EIS measurements. After each electrode preparation step, the electrode is removed and placed into solutions of 1 mM ferro/ferricyanide in 0.1 M NaClO_4_for analysis. Therefore, all the electrochemical measurements were conducted in the ferro/ferricyanide solution which eliminates the effect of any potential viscosity differences between the sample solutions. CV experiments were performed at a potential scan rate of 100 mV/s. Impedance spectra were recorded over a frequency range of 0.1–10^5^ Hz. A single sinusoidal AC voltage of 10 mV was superimposed on the open-circuit potential (typically 0.2 V vs. Ag/AgCl). The impedance was recorded and a Nyquist plot was obtained. A Randles circuit was used to fit the semi circular curves in order to calculate the charge transfer resistance (R_CT_). The change percentage of R_CT_ is defined as 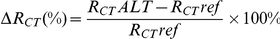
where R_CT_^ref^ and R_CT_^ALT^ is the charge transfer resistance before and after ALT binding.

## Results

### Selection and characterization of an ALT-binding peptide

**Figure 2:**
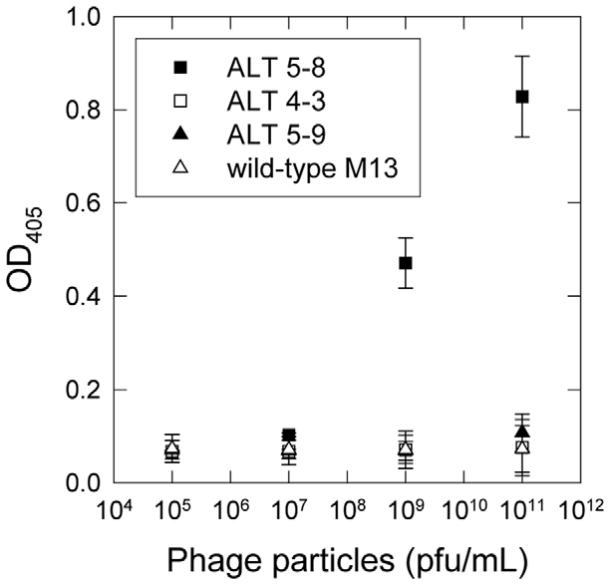
Characterization of the selected ALT binding phage clones using ELISA. ALT binding of the selected phage clones ALT5-8 (▪), ALT4-3 (□), ALT5-9 (▴) and M13 control phage (▵) with varying concentrations of phage particles ranging from 10^5^ pfu to 10^11^ pfu. All measurements were performed in triplicate and error bars represent standard deviations.

A large commercially available phage display library was biopanned over the immobilized ALT target protein. The yield of phage binding to the plate increased each round after the second (Table S1), even as the stringency of the selections was increased by the addition of Tween 20. Twelve phage clones randomly selected after the fifth round of selection were sequenced and the library was found to have converged (10/12) to one predominant sequence, ALT5-8, with a sequence of WHWRNPDFWYLK (Table 1). This clone along with two other sequences obtained in the sequencing (ALT4-3 and ALT5-9) were investigated using an ELISA assay to assess the affinity of the selected phage clones for immobilized ALT. The ALT4-3 and ALT5-9 clones were found to have a low affinity for ALT similar to that of the M13 control phage, However, the ALT5-8 clone exhibited a dose-dependent affinity for the ALT protein (Figure 2) and was selected for further characterization.

**Table 1:** Sequences of the 12 selected phage clones obtained following the fifth round of biopanning against ALT.

Name	Amino acid sequence	Frequency
ALT4-3	SNNLYPQRAVST	1/12
ALT5-8	WHWRNPDFWYLK	10/12
ALT5-9	LETEWDSLWYAP	1/12

**Figure 3:**
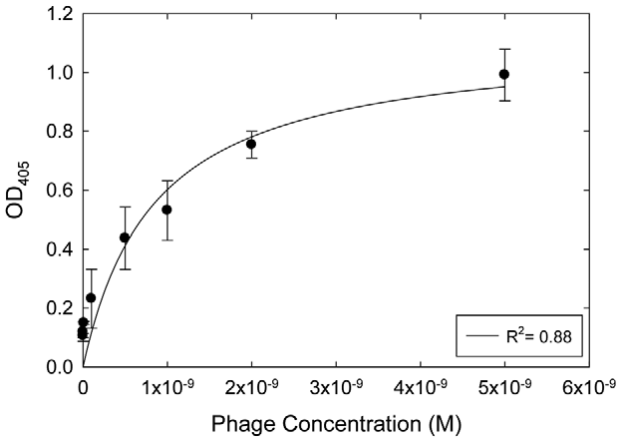
Determination of the apparent dissociation constant of ALT5-8 for ALT. ELISA experiments were performed in triplicate at eight different phage concentrations and the data were fit by a Langmuir isotherm (R^2^ = 0.88) in order to estimate the apparent equilibrium dissociation constant (K_d,app_ = 85±20 nM.).

Further ELISA experiments were performed with the ALT5-8 clone in order to determine the apparent affinity of the phage particles for the ALT protein. A fit of this data to the Langmuir isotherm (Figure 3) resulted in an apparent dissociation constant, K_d,app_ of 85±20 nM. Since the phage particles have approximately 5 copies of the peptide per particle, it is possible that the true affinity of the free peptide for the ALT protein will be different from the apparent value due to the loss of the avidity effect.

### Measurement of peptide inhibition constant, K_I_

The ALT target protein has a large surface area for peptide binding and the binding of the peptide in or near the active site of the enzyme could possibly affect the catalytic properties of the enzyme. Therefore, the impact of the peptide on the kinetics of the ALT enzyme was investigated. In the absence of the peptide, the kinetic data fit well to the bi-bi ping-pong equation (Eqn. 1) and the following kinetic parameters were obtained: k_cat_ = 5.8±0.2 s^−1^, K_M,alanine_ = 8.0±0.9 mM, and K_M,ketoglutarate_ = 0.090±0.030 mM (Figure S1) which are in agreement with previously published values for the ALT enzyme [38], [39], [40], [41].

**Figure 4:**
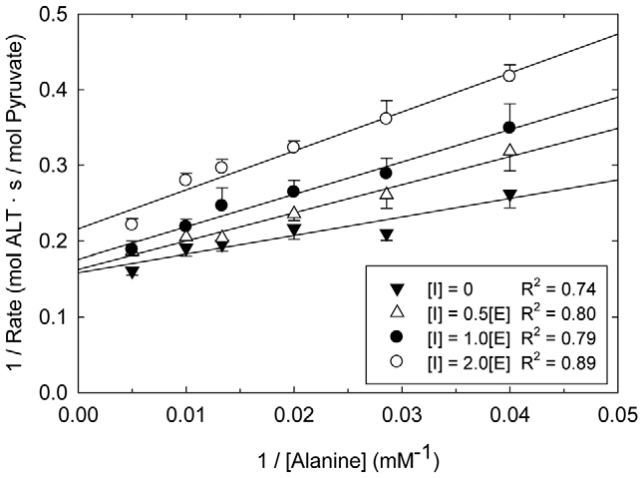
Lineweaver-Burk plots for the determination the of the ALT enzyme inhibition by the ALT5-8 peptide. Varying concentrations of peptide [I] were added as a function of initial ALT concentration [E]. All data were collected in triplicate and R^2^ values are shown for the best fit line for each inhibitor concentration. A K_I_ value of 71±17 nM was obtained by fitting the entire data set simultaneously to the bi-bi ping pong rate equation with competitive inhibition (Eqn. 2).

Lineweaver-Burk plots of pyruvate production at varying alanine concentrations are shown in Figure 4. The data suggests that the ALT binding peptide is a competitive inhibitor for the active site, with respect to alanine. The data were fit to the bi-bi ping-pong mechanism with competitive inhibition (Eqn. 2) using the k_cat_ and K_M_ values obtained in the absence of the peptide, and the inhibition constant for the ALT5-8 peptide was found to be K_I_ = 71±17 nM.

### Sensor preparation

**Figure 5:**
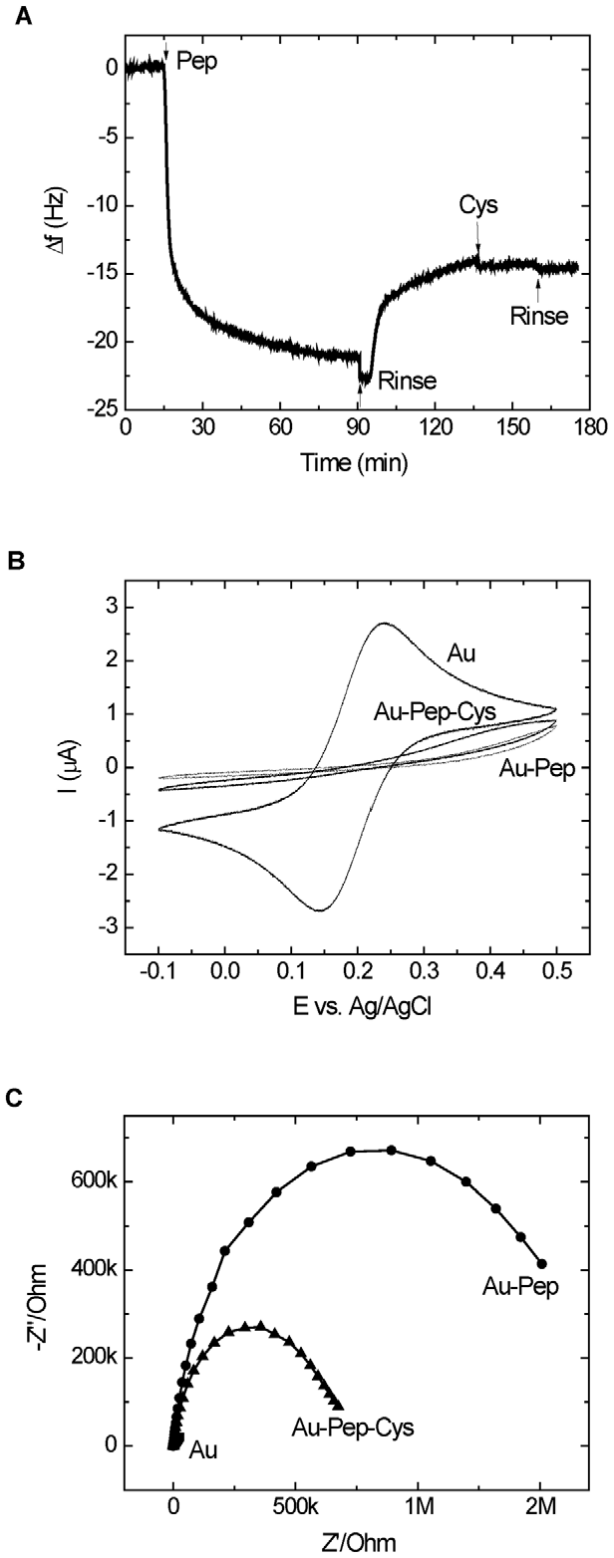
Sensor preparation. A) the change in frequency during the immobilization of peptide (Pep, 0.1 mM), rinsing (Rinse) and subsequent blocking with cysteine (Cys, 1 mM) on gold using QCM; B) CV of the bare gold electrode (Au), the electrode with the peptide (Au-Pep), and the blocked electrode (Au-Pep-Cys), C) EIS of bare gold (Au), the peptide modified electrode (Au-Pep) and the blocked electrode (Au-Pep-Cys) in a 1 mM solution of Fe(CN)_6_^4-/3-^ in 0.1 M NaClO_4_.

In order to use the phage selected ALT peptide in the development of an ALT biosensor, the peptide was modified with a C-terminal cysteine for immobilization on gold. The preparation of the sensor consisted of two steps: the immobilization of the peptide and blocking of empty sites with free cysteine. Both steps were monitored by QCM and electrochemical (CV and EIS) measurements as shown in Figure 5. The immobilization of the peptide resulted in a frequency drop in the QCM signal (Figure 5A) due to mass loading, a current decrease in the CV (Figure 5B), and an impedance increase in the EIS (Figure 5C) due to surface blocking. The abrupt changes in the QCM noted by arrows (Figure 5A) are due to pressure changes caused by valve switching. This phenomenon was observed in all the experiments. All the experiments were repeated three times with similar results each time. All three techniques show the successful immobilization of the peptides on the gold surface. Backfilling of the surface cysteine did not cause any mass change in QCM since both the amount and molecular weight of the blocking cysteine is small. However, the backfilling did result in a current increase and a prominent decrease in the impedance signal (smaller semi-circles). A similar effect was observed in our previous studies [16] and in studies of other modified surfaces [42]. The impedance decrease upon backfilling can be explained by the easier charge transfer through tunneling across the shorter cysteine. The charge transfer between Fe(CN)_6_^4-/3-^ and the electrode occurs either by tunneling of electrons through the surface self-assembled monolayer (SAM) (peptide or cysteine), or through defects (unblocked sites) on the surface [43]. The packing density of the immobilized peptide can be calculated using the Sauerbrey equation [16]:
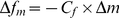
(3)where *Δf_m_* is the frequency change due to mass loading, *Δm* is the mass change at the QCM crystal surface and C_f_ = 0.0566 Hz/(ng/cm^2^) for a 5 MHz AT-cut crystal at 20°C. The frequency drop of the QCM due to peptide immobilization was about 21 Hz, which corresponds to a packing density of 2×10^−10^ mol/cm^2^ or 1.2×10^14^ molecule/cm^2^ based on a peptide molecular weight of 1851 g/mol. The surface density of a similar sized troponin-binding peptide immobilized on a gold surface has previously been reported to be 7.4×10^13^ molecule/cm^2^ [16]. Inamori *et al.* report a surface density of 1.8×10^12^ to 1.8×10^13^ molecules/cm^2^, depending on the attachment methods for an immobilized peptide on gold [44]. The average cross-section of each peptide was ∼0.8 nm^2^ based on the surface density, which indicates a dense packing mode of the immobilized peptides. The subsequent buffer rinse caused a small frequency increase (the vertical drop observed at the injection of buffer was caused by the sudden pressure change due to valve switching), possibly due to the removal of loosely bound peptide or the viscosity/density change of solutions.

### ALT sensor operation

**Figure 6:**
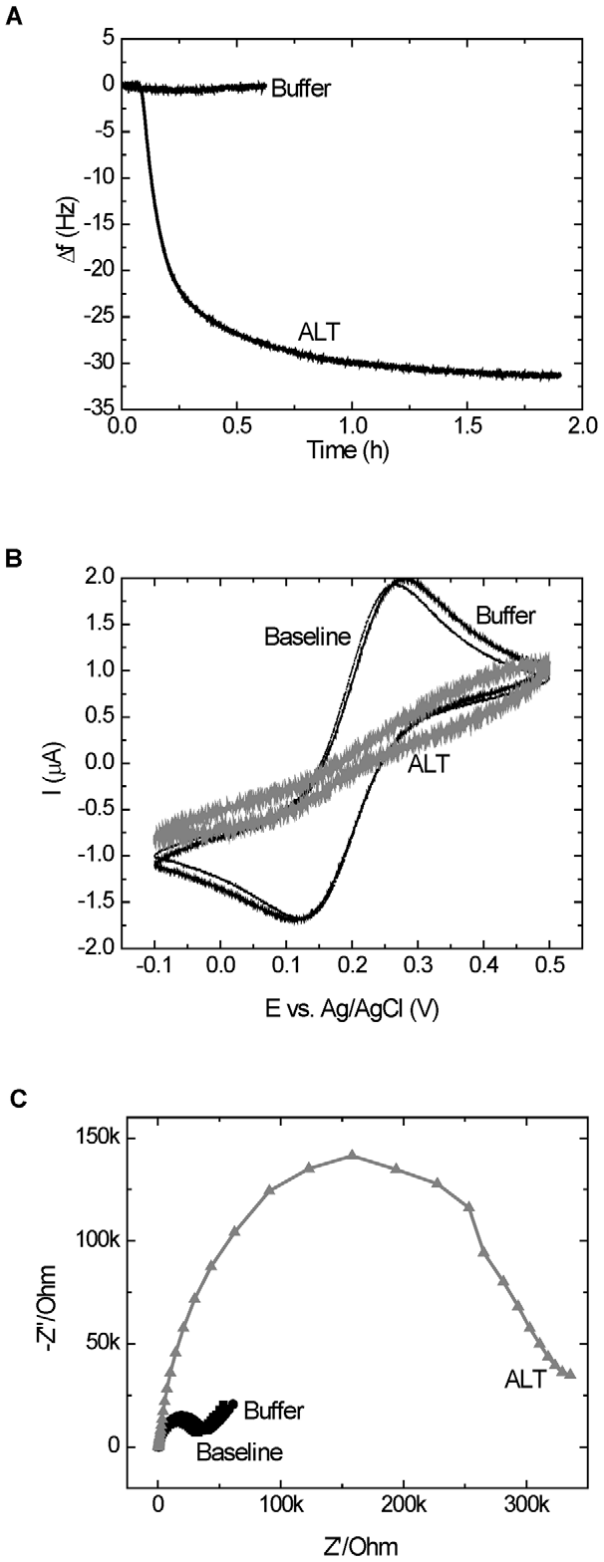
Sensor operation. A) QCM frequency change of Au-Pep-Cys electrode in Buffer alone and in 10 µg/mL ALT, B) CV of Au-Pep-Cys electrode before (Baseline) and after Buffer alone or ALT binding, C) EIS of Au-Pep-Cys before (Baseline) and after Buffer alone or ALT binding. After incubation in Buffer or 10 µg/mL ALT, the electrodes were transferred to 1 mM solution of Fe(CN)_6_^4-/3-^ in 0.1 M NaClO_4_ for the EIS measurements.

QCM, CV and EIS measurements validated the formation of the peptide-based sensing layer. The same techniques were used to demonstrate the affinity of the immobilized peptide for ALT, as shown in Figure 6. Upon binding of ALT by the immobilized peptide, a frequency decrease (*Δf* = -31 Hz) in QCM (Figure 6A), a current decrease in CV (Figure 6B), and a large impedance increase in EIS (Figure 6C) were observed. No change was observed for the buffer control in all three cases. All three techniques indicate binding of ALT to the immobilized peptide.

**Figure 7:**
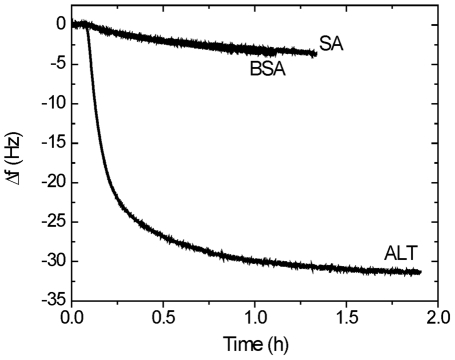
Specificity of the ALT biosensor. The shift in QCM frequency in response to injections of SA, BSA or ALT on the Au-Pep-Cys modified crystal. The concentrations of all three solutions were 10 µg/mL.

To begin to study the selectivity of the peptide, similar experiments were performed with streptavidin (SA) and bovine serum albumin (BSA). Figure 7 shows the frequency change of Au-Pep-Cys binding to SA, BSA and ALT. A small frequency change of about -3 Hz was observed upon addition of SA and BSA, while a frequency change of -31 Hz was observed with ALT. The small frequency change seen with the control proteins might be due to nonspecific binding of SA and BSA with unoccupied sites on the surface or non-specific interactions with the peptide.

**Figure 8:**
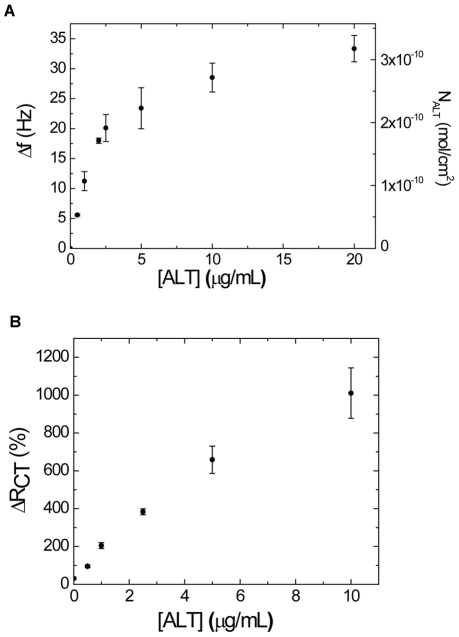
Response curves of ALT binding. Measurements were made using QCM (shown as absolute value of frequency change) (A) and EIS (B). Each run was performed using a single electrode with successive tests in ALT solutions from low to high concentration. The apparent amount of bound ALT (N_ALT_) for the QCM measurements was calculated using Eqn. 3 and is shown on the right ordinate. Error bars represent standard deviations obtained from triplicate measurements.

Although all three techniques demonstrate a signal change upon the binding of ALT, the CV technique is a poor candidate for quantitative measurement since the detection range is limited by the fact that the current decreases upon target binding. For QCM and EIS, response curves over a range of ALT concentrations are shown in Figure 8. Both QCM (Figure 8A) and EIS (Figure 8B) show a quantitative response to a range of ALT concentrations and thus can be used as a sensing technique in quantitative ALT measurements. The LOD obtained by QCM is 60 ng/mL, while EIS had a LOD of 92 ng/mL. The sensitivity of the QCM system was 8.9±0.9 Hz/(µg/mL), while the sensitivity of the EIS system was 142±12 impedance percentage change %/(µg/mL).

Based on the QCM response curve, the kinetic binding constants can be deduced. As described previously [16], the equilibrium dissociation constant, *K_d_*, can be obtained from a plot of frequency change, *Δf_eq_*, vs. *Δf_eq_*/*C*. Here, a dissociation constant of *K_d_* = 2.01
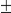
0.06 µg/mL or 20.1
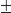
0.6 nM was calculated for ALT binding on QCM.

## Discussion

Our overall goal is to develop a streamlined platform for the rapid development of new biosensors that can be used to detect virtually any desired protein target. Phage display is a commonly used method for identifying new binding motifs, and commercially available kits make this process broadly accessible [4], [7]. Biopanning procedures are well documented, and new binding peptides can usually be identified in a short period of time (1–2 weeks). There are several detection methods available that can be used to create a biosensor from a recognition peptide. We have chosen electrochemical techniques since they are generally simple, inexpensive, and can be readily incorporated into microfluidic devices. In this manuscript, we demonstrate this process from start to finish to create a new biosensor for the detection of ALT.

ALT is an enzyme, and one approach for its detection is to monitor its enzymatic activity [29], [35], [36]. This approach can be very sensitive, but it is not a general platform for biosensor development, and this approach needs to be redeveloped for each new enzyme to be detected. We have chosen instead to directly detect the presence of the protein using an evolved binding peptide, and in this way the detection method is generic and does not depend on the enzymatic activity of the target protein. The phage display library converged to a new binding sequence after 5 rounds of biopanning (ALT5-8) and this peptide exhibited nanomolar affinity for ALT when displayed on phage particles. We further explored whether the peptide was binding near the active site of the enzyme through enzyme inhibition studies. The peptide was found to be a competitive inhibitor for ALT with a nanomolar inhibition constant. However, the enzyme was still quite active even when the peptide to protein ratio was at 2∶1. Therefore, it is possible that the enzyme is still active in the bound state, and future work will be necessary to determine if a biosensor can be created based on detecting the bound ALT activity instead of monitoring the mass change that occurs upon binding of the enzyme.

Another concern with this approach is that peptides identified using phage display may lose affinity for their target when they are displayed on a different platform. There are, on average, 5 copies of the ALT5-8 peptide on the surface of the M13 phage, and this avidity effect gives rise to an apparent nanomolar dissociation constant. This proved not be a significant problem as, when peptides were immobilized on a solid surface, a true dissociation constant for the ALT binding peptide remained in the nanomolar scale range.

As we previously found with Troponin I, EIS appears to be the optimal method for the electrochemical detection of the bound target [16]. The procedure is straightforward, and high sensitivities and low LOD values are obtained. The only downside to the EIS technique is the need to measure the impedance signal in the presence of a redox couple after the sample has been exposed to the peptide sensing layer. But, the EIS technique produces a larger linear sensing range than can be obtained with QCM and it is more robust than QCM as it is less sensitive to environmental perturbations.

The new ALT biosensor made from the ALT5-8 peptide coupled with EIS detection had a LOD (0.092 µg/mL) close to the normal level of ALT in human blood (0.1–0.7 µg/mL) [29]. Although we have demonstrated that the ALT5-8 peptide is selective for ALT as compared to BSA and SA, further work will be required to explore the performance of the biosensor in real samples such as tissue culture media or plasma, and in the presence of other potential interferents.

Although this approach will require further development before it can be used to create a biosensor for clinical use, the use of unstructured peptides obtained via phage display coupled with electrochemical detection methods has several advantages compared to other biosensor platforms described in the literature. Alternative recognition elements such as antibodies are time consuming and expensive to produce, and aptamer-based sensors can require complex sample labeling and modification techniques [45]. The unstructured peptides used in this approach can be easily selected against virtually any target and are inexpensive to synthesize, but they may suffer from poor selectivity in complex solutions. Several transduction techniques are also available, especially optical methods such as surface plasmon resonance (SPR) [46] and Förster resonance energy transfer (FRET) [47], but EIS has the potential to be one of the most powerful and cost-effective tools for biosensor development [48].

In summary, we have developed a general method for the creation of new biosensors using short synthetic peptides obtained via biopanning of a phage displayed library. The whole process includes: target selection and immobilization, phage display to select binding peptides, peptide synthesis with a terminal thiol, QCM in-situ monitoring of peptide immobilization, and sensor detection using electrochemical techniques (Figure 1). We have demonstrated this approach by creating a new biosensor for a well-known biomarker for hepatocellular toxicity, ALT. The new biosensor had a LOD value just below the physiological concentration of the target protein in human blood. Since both phage display and EIS are widely used techniques, this general approach to biosensor development is straightforward and can be readily applied for the development of biosensors to detect almost any desired protein target.

## Supporting Information

Figure S1 **Uninhibited ALT enzyme kinetic data.** Kinetic experiments were performed in a coupled assay where the pyruvate generated by the ALT reaction was reduced to lactate by LDH with the concomitant oxidation of NADH. Varying concentrations of L-alanine (A) and α-ketoglutarate (B) were used, and the data were fit to the bi-bi ping-pong mechanism (Eqn. 1) using non-linear regression software (SigmaPlot). The Michaelis constants for L-alanine and α-ketoglutarate were determined to be 8.0±0.9 mM and 0.090±0.030 mM respectively with k_cat_ = 5.8±0.2 s^−1^. All data were collected in triplicate and error bars represent standard errors.
(TIF)

Table S1 **Enrichments obtained by selecting the phage displayed peptide library over immobilized ALT.**
(DOCX)
