# Supplementary figures and images for: Rapid Development of New Protein Biosensors Utilizing Peptides Obtained via Phage Display
Source: PLoS One. 2011 Oct 7;6(10):e24948. doi: 10.1371/journal.pone.0024948 (PMC3189179; doi:10.1371/journal.pone.0024948)

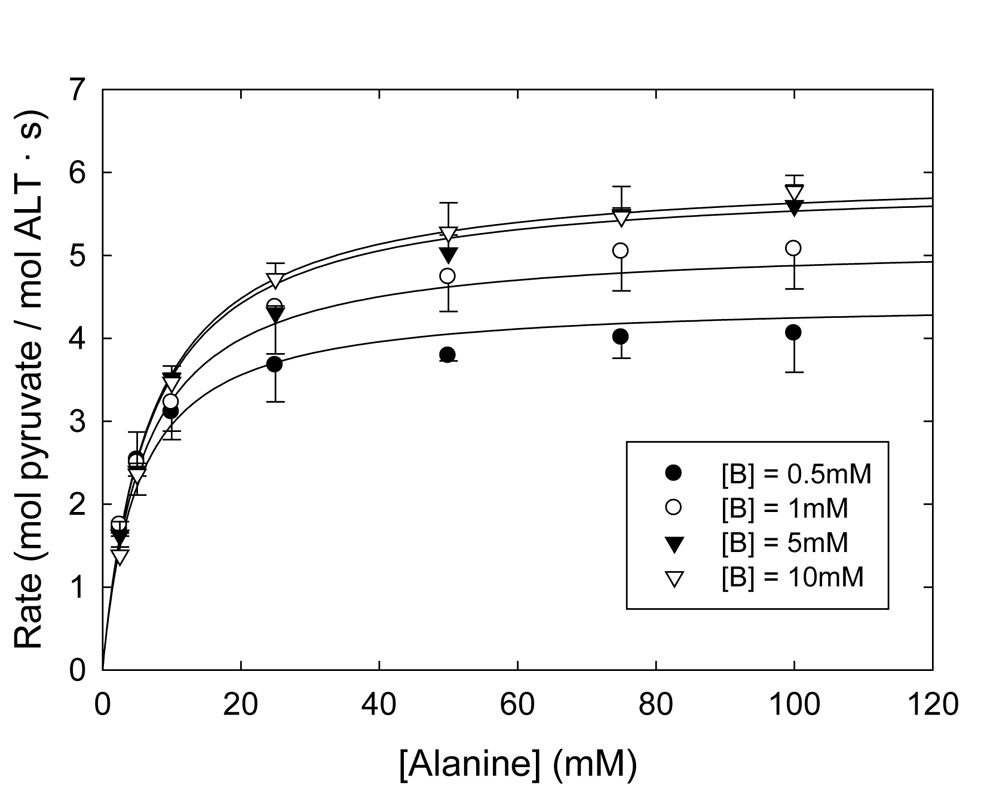

Supplement: Figure S1 — Uninhibited ALT enzyme kinetic data. Kinetic experiments were performed in a coupled assay where the pyruvate generated by the ALT reaction was reduced to lactate by LDH with the concomitant oxidation of NADH. Varying concentrations of L-alanine (A) and α-ketoglutarate (B) were used, and the data were fit to the bi-bi ping-pong mechanism (Eqn. 1) using non-linear regression software (SigmaPlot). The Michaelis constants for L-alanine and α-ketoglutarate were determined to be 8.0±0.9 mM and 0.090±0.030 mM respectively with kcat = 5.8±0.2 s−1. All data were collected in triplicate and error bars represent standard errors. (TIF) [file pone.0024948.s001.tif]
