# Supplementary material for: Rapid Development of New Protein Biosensors Utilizing Peptides Obtained via Phage Display
Source: PLoS One. 2011 Oct 7;6(10):e24948. doi: 10.1371/journal.pone.0024948 (PMC3189179; doi:10.1371/journal.pone.0024948)
Supplement: Table S1 — Enrichments obtained by selecting the phage displayed peptide library over immobilized ALT. (DOCX) [file pone.0024948.s002.docx]

**Table S1**. **Enrichments obtained by selecting the phage displayed peptide library over immobilized ALT.**

| Biopanning Round | Tween 20 (v/v %) | Phage Input Titer (pfu/mL) | Phage Output Titer (pfu/mL) | Yield (%) |
| --- | --- | --- | --- | --- |
| 1^st^ | 0.1 | 1.5 x 10^11^ | 1.6 x 10^6^ | 1.1 x 10^-3^ |
| 2^nd^ | 0.3 | 0.7 x 10^11^ | 0.1 x 10^6^ | 1.4 x 10^-4^ |
| 3^rd^ | 0.5 | 2.1 x 10^11^ | 1.4 x 10^6^ | 6.7 x 10^-4^ |
| 4^th^ | 0.5 | 1.9 x 10^11^ | 6.2 x 10^8^ | 0.3 |
| 5^th^ | 0.5 | 0.6 x 10^11^ | 1.5 x 10^10^ | 25 |
